# Impact of Dental Treatment, Including Multiple Extractions, Under General Anaesthesia on Children’s Oral Health-Related Quality of Life: A Prospective Study

**DOI:** 10.3390/dj13050202

**Published:** 2025-05-01

**Authors:** Haneen Baty, Ibtesam Alzain, Medhat Abdulla, Khlood Baghlaf

**Affiliations:** 1 Ministry of Health, Tabouk 47913, Saudi Arabia; hahmedbaty@stu.kau.edu.sa; 2 Pediatric Dentistry Department, King Abdulaziz University, Jeddah 22254, Saudi Arabia; kbaghlaf@kau.edu.sa; 3 Department of Pediatric Dentistry, Alexandria University, Alexandria 5372066, Egypt; medhat.abdallah@alexu.edu.eg

**Keywords:** oral health, dental caries, teeth extraction, quality of life

## Abstract

**Background/Objectives:** Children who undergo several dental extractions with general anesthesia (GA) may face considerable changes in their oral health-related quality of life (OHRQoL), but there is a lack of research on this issue in the Middle East. **Aim:** This study aimed to assess how the number of dental extractions performed under general anaesthesia due to caries impacts the oral health-related quality of life (OHRQoL) in children aged from three to six years. **Methods:** This prospective, single-center cohort study included parents of children aged 3–6 years undergoing dental treatment under general anaesthesia in Jeddah, Saudi Arabia. Parents completed the validated Arabic Early Childhood Oral Health Impact Scale (A-ECOHIS) before and after treatment to assess changes in OHRQoL. The number of extractions was recorded, and patients were grouped based on extraction frequency: high (≥mean) and low (<mean). Pearson’s correlation was used to evaluate the relationship between the number of extractions and the effect size of OHRQoL. **Results:** Ninety-three participants met the inclusion criteria and agreed to participate in this study. The mean age of the children was 4.88 years (SD ± 1.06). The most common procedure performed was extraction, with a mean of 5.34 (SD ± 5.53), followed by stainless-steel crown application, with a mean of 4.03 (SD ± 2.01). No correlation between the number of extractions and the effect size in the change in OHRQoL was noted (Pearson r = −0.002, *p*-value = 0.98). **Conclusions:** Dental extractions were the most common procedure performed under GA. While multiple extractions showed no significant association with the effect size of OHRQoL, overall treatment under GA led to significant improvement. These findings highlight the need for timely intervention, parental education, and comprehensive treatment protocols. Utilizing tools like ECOHIS may aid in prioritizing high-risk cases and optimizing resource allocation in pediatric dental care.

## 1. Introduction

Dental caries remains the most prevalent chronic infectious disease among children [1], with Early Childhood Caries (ECC) typically presenting as either cavitated or non-cavitated lesions and/or missing teeth in children under five, often linked to poor dietary habits and low socioeconomic status [2]. Characterized by rapid progression, ECC poses a significant public health concern due to its effects on children’s development. Symptoms associated with ECC—such as pain, infection, and tooth loss—can hinder nutrition and academic performance. If left untreated, ECC may result in pulpal inflammation and extraoral swelling, further reducing quality of life by impacting eating, speaking, and social interactions [3,4]. In Saudi Arabia, a recent cross-sectional study found that 65.6% of children aged 6, 12, and 15 had dental caries [5]. The study also demonstrated that the prevalence of dental caries in primary teeth was 72%, compared to 61% in permanent teeth. Furthermore, the rates of missing or filled teeth among participants were 9% and 20%, respectively.

The dental management of these young patients presents numerous challenges for both clinicians and parents [6,7]. Behavioral management is particularly challenging in preschool-aged children due to developmental limitations and restricted communication, especially in cases requiring complex procedures [8]. Various strategies have been developed to address this, ranging from non-pharmacological approaches—such as verbal communication, voice control, and positive reinforcement—to pharmacological methods, including sedation and general anesthesia [9,10]. While many cases can be managed in a conventional dental setting, some young patients—particularly those with extensive ECC—require advanced interventions such as nitrous oxide inhalation, conscious sedation, or general anesthesia (GA) to enable safe and effective treatment [11].

Oral health-related quality of life (OHRQoL) encompasses many aspects used to evaluate how oral health affects a child’s overall, mental, and physical well-being [12]. Various tools have been used to assess Oral Health-Related Quality of Life (OHRQoL) following dental treatment under general anesthesia. These include the Family Impact Scale (FIS), the Parental-Caregivers Perceptions Questionnaire (P-CPQ), and the Early Childhood Oral Health Impact Scale (ECOHIS) [13,14]. These methods generally involve questionnaires completed by parents or children to detect changes in OHRQoL before and after treatment. Many studies have demonstrated that these OHRQoL instruments are both valid and reliable [15,16].

Recent research assessing the impact of dental treatment under GA on children’s OHRQoL indicates that their quality of life improves following such treatment [8,17,18]. Many of these longitudinal studies evaluated the post-treatment OHRQoL within a month after the procedure; however, children may still be experiencing discomfort from the extractions during this period. Furthermore, none of these studies has examined the relationship between the number of extractions and their impact on children’s OHRQoL following comprehensive dental treatment under GA in the Middle East.

In the United Kingdom, studies have examined how dental extractions affect children’s OHRQoL, showing significant improvement following extractions under GA [19,20]. However, there is a lack of research on how multiple extractions (three or more) under GA impact children’s OHRQoL in the Middle East. Thus, this study aimed to evaluate the effects of multiple extractions under GA on children’s OHRQoL. The null hypothesis states that multiple extractions performed under dental general anesthesia do not impact children’s oral health-related quality of life.

## 2. Materials and Methods

### 2.1. Study Design

This study employed a prospective, single-center cohort design and was conducted at King Abdulaziz University Dental Hospital (KAUH) in Jeddah. The research followed the Declaration of Helsinki, and ethical approval was obtained from the ethics committee at King Abdulaziz University Faculty of Dentistry (REC-FD 03-02-22) on 20 October 2022. Consent forms were collected from all participants in this study.

### 2.2. Sampling

The sample size was determined using G.power software (3.1.9.4). The significance level of 0.05 and a power of 80% were used as a base for calculation. The outcome was the change in the ECOHIS scores, and the effect size measured was 0.423 [8]. It was estimated that 88 sample sizes were adequate to complete this study. A convenience sampling technique was used to recruit parents of three to six-year-old children who received dental treatment under GA. The very young or uncooperative children, referred from paediatric dentistry clinics for dental treatment under GA due to extensive dental needs, were considered in this study.

### 2.3. Selection Criteria

All parents of healthy children scheduled to receive dental treatment under GA, including multiple teeth extractions (three or more), were invited to participate in this study.

The inclusion criteria for participants were as follows: children whose parents agreed to participate in this study, children aged between three and six years, medically fit participants (classified as ASA I by the American Society of Anesthesiologists), and children requiring multiple extractions (three or more teeth). The exclusion criteria included children whose parents refused to participate, medically compromised children (classified as ASA II or greater), and patients without planned extractions in the treatment plan or those requiring fewer than three teeth to be extracted.

### 2.4. Data Collection Procedure

Parents of children referred to the day surgery unit for full mouth rehabilitation, which included multiple tooth extractions under GA, were identified as potential participants for this study. One week prior to the GA day, during the treatment planning visit at the postgraduate clinic, parents of eligible children were approached and provided with an invitation letter along with detailed information about this study. If a parent agreed to participate, they received a consent form to sign and a pre-operative questionnaire. Additionally, the consent forms included a section ensuring that all data collected from participants would remain strictly confidential. The consent form was available in both Arabic and English. Any questions regarding the questionnaire were addressed at that time.

### 2.5. Questionnaire

This study employed a validated tool to evaluate oral health-related quality of life (OHRQoL). The Early Childhood Oral Health Impact Scale (ECOHIS) questionnaire was used to assess OHRQoL in children both before and after dental treatment under general anesthesia (GA). The ECOHIS questionnaire is mainly designed for preschool-aged children and consists of two primary sections with a total of 13 questions directed at both parents/caregivers and the child. These sections include the child impact section (nine questions) and the family impact section (four questions). Responses are rated on a 6-point scale: 0 = never; 1 = hardly ever; 2 = occasionally; 3 = often; 4 = very often; and 5 = do not know. The total score can range from 0 to 52 (0–36 for the child section and 0–16 for the family section). This questionnaire has been translated into several languages, including Arabic, and its reliability and validity have been assessed in the Arabic version of A-ECOHIS.

The same parent completed the questionnaire at both time points. Additionally, participants completed a questionnaire concerning oral health behavior, which included demographic and dental health-related questions from a validated oral health and behaviour questionnaire (OHBQ).

### 2.6. Follow-Up Visit

Six weeks after the dental treatment under general anaesthesia, patients were scheduled for a follow-up visit. After the examination, the same parents were asked to complete the post-operative questionnaire. If parents missed the follow-up appointment, they were contacted by phone to gather their responses. Details of the dental treatment, including the number of extractions, restorations, stainless-steel crowns, and pulp therapies (such as pulpotomy, pulpectomy, lesion sterilization and tissue repair, direct pulp capping, and indirect pulp capping), were reviewed from the progress notes and confirmed during the follow-up visit via clinical examination.

### 2.7. Statistical Analysis

The data collected were entered and analyzed using SPSS Statistics for Windows, version 22.0 (SPSS Inc., Chicago, IL, USA). All data were anonymized and stored on a password-protected computer. Categorical demographic variables were reported as percentages and frequencies, while means and standard deviations were computed for continuous variables. The primary outcome was the effect size of OHRQoL. The effect size was also calculated as follows: (34,35) [21] ES = Mean baseline score—mean follow-up score/SD of mean baseline score.

The mean was used to categorize each dental procedure based on its frequency. Pearson’s correlation was used to assess the correlation between the mean effect size of OHRQoL and the mean number of extractions.

Several extractions were classified into two categories: high if they were equal to or greater than the mean, and low if they were less than the mean. Additionally, other dental procedures, such as pulp therapy, restorations, and stainless-steel crowns (SSCs), were categorized based on the mean scores. Moreover, a bivariate analysis was conducted between the various categories of the effect size of OHRQoL and the different categories of dental procedures using the Chi-square test, with *p* ≤ 0.05 considered statistically significant.

A linear regression model was used, where the outcome was the effect size of OHRQoL. The factors included extraction categories, restoration categories, and pulp therapy categories. The SSCs categories were removed from the model as they have a similar effect to pulpotomy categories. The covariates included sex, pain, and income.

## 3. Results

### 3.1. Demographic Variables

Of the one hundred five participants who met our inclusion criteria, only 93 agreed to participate and attended the six-week follow-up appointment. Nearly half of the participants were female, with 51 (53%) compared to 42 (45%) males, and the mean age was 4.88 (SD ± 1.06). Table 1 presents the demographic variables of the participants. The most common age range for parents was between 31 and 40 years, accounting for 57 (60%), while 65 (66%) had a college or postgraduate degree. The least represented parental age group in our study was from 20 to 30 years, comprising 14 (12.9%), and only 12 (13%) reported having less than a high school level education. Eighty-one (83.9%) of the parents in our study were married and lived with their children. Our study found that 39 (40.9%) of the parents reported a high monthly income, while 33 (33%) reported a medium monthly income.

### 3.2. Effect Size and Quality of Life

Table 2 presents the effect size of the improvement in OHRQoL, categorized into three levels: low, moderate, and large effect size. More than half of the participants, 48 (51.6%), experienced a large effect size in OHRQoL, while 17 children (18.3%) exhibited a moderate effect size, and 28 participants (30%) had a low effect size.

### 3.3. Dental Procedures Performed Under General Anaesthesia

The most common procedure performed under general anaesthesia was extraction (5.34, SD ± 5.53), followed by SSCs (4.03, SD ± 2.01), restorations (2.73, SD ± 2.41), and, finally, pulp therapy (2.17, SD ± 2.29). Tooth extraction was found to be the most common procedure administered under general anaesthesia. More than half of the children, 53.8%, had more than five teeth extracted, while 46.2% had from three to four teeth extracted.

### 3.4. Association Between Multiple Extractions and Effect Size of OHEQoL

A Pearson correlation test was conducted to measure the effect of multiple extractions on the mean effect size of OHRQoL and multiple extractions. No correlation between the number of extractions and the effect size in the change in OHRQoL was noted (Pearson − 0.002, *p*-value = 0.98).

Table 3 shows the link between the number of extractions (categorized as 3–4 teeth extracted and more than five teeth) and the various effect sizes in the change in the OHRQoL category (categorized as low, moderate, and large). The Chi-square test revealed no statistically significant differences (*p*-value = 0.32). However, children with more than five extractions reported a significant change in the effect size of OHRQoL. Furthermore, children with more SSCs and pulp treatments reported a large effect size of OHRQoL; however, the difference was not statistically significant (*p*-value = 0.85, 0.30, respectively).

Table 4 presents the results of the regression model. The findings indicated that children with a history of dental pain had a significant association with a large effect size on OHRQoL (B: 0.26; *p* = 0.04). No significant associations were observed between extraction categories and the effect size of OHRQoL.

## 4. Discussion

This study examined how multiple dental extractions (due to dental caries) performed under general anaesthesia affected the effect size of OHRQoL among children aged from three to six years. This prospective longitudinal study employed a convenient sampling technique to assess OHRQoL at baseline and at six weeks post-treatment. To our knowledge, no study in the Middle East has investigated the influence of multiple GA extractions on a child’s OHRQoL at such a young age (ages 3–6). This study evaluated the impact of multiple dental extractions under general anesthesia on the quality of life in young children. The study design did not include a comparison between non-extraction and extraction cases, as such a comparison is nearly impossible due to the nature of the patient cohort studied. Patients scheduled to undergo treatment under GA are high caries risk patients and almost always require extractions, with only 3–5% needing routine treatment. Therefore, such a comparison is not feasible within the sample of patients studied in our research.

To ensure the reliability of the results and avoid potential confounding factors, children with underlying medical conditions were excluded from the sample. This allowed for a more accurate assessment of the effects of dental treatment alone, independent of systemic health issues. Although this study did not specifically aim to explore social fragility, it is important to note that the sample included children from diverse social backgrounds. The research was conducted in a governmental hospital setting, where all patients receive free treatment under general anesthesia. This setting helped ensure that access to care was not influenced by socioeconomic status, allowing for a more representative and equitable sample. While minimally invasive strategies are a critical aspect of managing paediatric dental patients, especially those with health or social vulnerabilities, the current study focused on children who had already been referred for general anesthesia due to extensive treatment needs and behavioral management challenges.

The 6-week post-treatment follow-up period was chosen because it falls within the most commonly selected follow-up intervals, which, according to a 2018 meta-analysis, range from 4 weeks to 3 months [16]. Since the literature shows no difference in treatment effects on the OHRQoL when assessed after 2 weeks compared to 3 months, the 6-week follow-up period appears justifiable [22]. A challenge during data collection for longitudinal studies is the response rate from parents and children after surgery, which was effectively addressed by increasing the sample size from 88 to 93.

The tools most commonly used to evaluate the OHRQoL are the ECOHIS and the P-CPQ [20,23]. This investigation selected a relevant instrument based on the age group and the availability of a validated Arabic version. A prior study [24] validated the Arabic version of the ECOHIS. One of the main challenges for parents and caregivers is the expense related to complex treatment plans, particularly those requiring GA. By examining the changes in OHRQoL as outcomes, we may influence the perspectives of parents and caregivers, helping patients and their families make more informed treatment decisions. However, all children were recruited from a single government hospital, and the parents or caregivers incurred no costs.

Furthermore, since the included age group was quite young, using an instrument that involved a parent proxy was recommended. This is because younger children may not fully understand or express themselves, which is another reason the ECHOIS was a better fit [25]. A study conducted in Saudi Arabia explored how fathers and mothers differ in their perceptions of the oral health-related quality of life (OHRQoL) in young children aged from 2.1 to 5.7 years [26]. The results indicated that mothers were more consistent than fathers in assessing their children’s OHRQoL. Additionally, the analysis of the current study showed that fathers were more inclined to respond with “don’t know”, suggesting they had a lower level of accurate knowledge about their children’s oral health compared to mothers [26].

Six weeks following dental treatment under GA, more than half of the participants (51.6%) had a large effect size in the change of OHRQoL. Consistent with earlier studies, OHRQoL improved significantly with dental treatment under GA after four weeks, three months, and a year [9,19,21,27]. Therefore, the null hypothesis is rejected.

According to our research, extractions were the most common dental procedure performed under general anaesthesia, followed by the insertion of SSCs and restorations. In line with the AAPD guidelines, aggressive treatment of dental caries under general anaesthesia is implemented to ensure better treatment outcomes and reduce the incidence of dental general anaesthesia failures [28]. Conversely, a cross-sectional study conducted in the United Arab Emirates evaluated the OHRQoL of 173 preschoolers following a dental treatment using the ECHOIS and found that pulp therapy was the most often performed procedure. The next most prevalent procedures were SSCs, restorations, and extractions [23].

In our findings, children who had more than five extractions reported a substantial effect size of OHRQoL. However, no significant differences existed between the effect size of OHRQoL and the number of extractions. AlBader (2019) reported that a study evaluating OHRQoL in children aged from five to eight years old, utilizing a Child-Centered Caries-Specific Quality of Life (CARIES-QC) questionnaire on 35 participants, demonstrated a significant improvement following extractions under GA [19]. At the first follow-up at one month, the effect size of OHRQoL was 0.93, and the second follow-up at three months was 1.66. They found a statistically significant moderate correlation between the total number of teeth extracted and the follow-up CARIES-QC score, r = 0.453, *p* < 0.05 [19].

Furthermore, our study’s regression analysis revealed a non-significant association between the extraction categories and the effect size of OHRQoL. This contradicts earlier research that showed that OHRQoL was associated with the number of teeth treated under GA in preschool children [19,25]. The regression analysis of our study showed that children who reported a history of dental pain had a significant association with a large effect on OHRQoL. This can be attributed to the fact that pain was the most common complaint, and, once it was removed, the quality of life significantly improved.

Another study compared OHRQoL in children aged from zero to five after teeth extraction to pulpectomy treatment [25]. The results revealed a considerable improvement in both treatments (extractions and pulpectomies), with higher total ECOHIS scores in the extraction group, indicating a worsening in OHRQoL [25]. This could be due to the repercussions of early primary tooth loss, such as difficulty chewing food, particularly in the first few weeks after extraction [25]. The overall ECOHIS was found to be higher when patients extracted their posterior teeth; this has been connected to postoperative problems such as agitation. As a result, total ECOHIS ratings have fallen greatly when compared to previous treatments, while all OHRQoL levels have dramatically improved [25].

Several strengths were found in the current study, including the use of a validated (ECOHIS) questionnaire to assess OHRQoL. To our knowledge, this study was the first in the Middle East to evaluate the impact of several extractions under GA on a child’s OHRQoL. The current study followed a strict methodological plan to ensure consistency, accuracy, and dependability. These factors work together to strengthen and credibly support this study’s conclusions. The same parent completed the questionnaire before and after GA to ensure consistency. This method removes unpredictability that could result from different people’s interpretations or biases in answering the questions.

However, no study is without limitations. One of the primary drawbacks of utilizing a convenience sample is sampling bias (or selection bias). However, randomization was not attainable due to this study’s design. Furthermore, the data were collected from a single institute, which restricts the generalization of the conclusions. Additionally, the 6-week follow-up period may capture only short-term changes that are particularly relevant to patients’ immediate postoperative experiences, such as relief from pain or discomfort. However, these improvements may primarily reflect transient symptom relief rather than sustained enhancements in OHRQoL. Longer-term outcomes, including functional adaptation, changes in dietary habits, and the potential need for further dental or medical interventions, may arise well beyond the initial postoperative phase and, therefore, require a longer follow-up period. This study investigated the influence of several extractions on OHRQoL; however, no analysis was conducted to compare the extraction of anterior and posterior teeth. Due to the limited research specifically examining the impact of dental extractions on oral health-related quality of life (OHRQoL), this study focused on the number of teeth extracted as an initial step toward addressing this gap. We acknowledge that additional clinically relevant variables—such as the location of the extracted teeth (anterior vs. posterior), the presence or absence of restorative or prosthetic rehabilitation, and associated functional implications—may significantly influence OHRQoL outcomes. These factors merit further investigation and will be valuable considerations for future research.

## 5. Conclusions

Based on the study results, the following conclusions have been drawn:

1. Dental treatment under GA leads to a significant improvement in children’s OHRQoL.
2. The most common procedure performed under GA was dental extraction
3. Multiple extractions have no significant association with childrens’ OHRQoL effect sizes after dental treatment under GA.
4. Children with a high number of extractions (≥5 teeth) reported a large effect of change on OHRQoL.

### 5.1. Recommendations

- Educate parents about the oral health-related quality of life (OHRQoL) of patients and its impact on the overall, social, and economic aspects of both children’s and parents’ lives.
- Advocate for the implementation of comprehensive treatment protocols under GA, particularly for the extraction of problematic primary teeth.
- Highlight the importance of oral health practices for parents.
- Encourage the use of ECOHIS to help prioritize the waiting list for general anesthesia.

### 5.2. Future Direction

It is recommended that future longitudinal studies involve a larger sample size, consider multiple centers, and include longer follow-up periods to evaluate the effects of full mouth rehabilitation under GA, including multiple extractions, on children’s OHRQoL.

Furthermore, future studies can assess the impact of anterior and posterior extractions, the presence or absence of prosthetic or restorative replacement, and functional impacts on children’s oral health-related quality of life (OHRQoL). Future studies can use additional proxy measures to assess children’s perceptions of preschool teachers. Moreover, further research is warranted to investigate the effectiveness of less invasive alternatives in children with various medical and social fragility levels.

## Figures and Tables

**Table 1:** Descriptive characteristics of children and their parents.

Demographic Data		Frequency (*n*)	Percentage (%)
**Parents’ ages**	20–30	14	12.9
	31–40	57	60.2
	>40	22	23.7
**Parents’ education**	No education/Primary school/Middle school	12	13
	High school/diploma	16	17.2
	College/post-grad	65	66.6
**Marital status**	Married	81	83.9
	Divorced/widowed	12	12.9
**Child’s residence**	Both parents	81	83.9
	Father	3	3.2
	Mother	9	9.7
**Parents’ monthly income**	<7000	21	22.6
	7000–10,000	33	33.3
	>10,000	39	40.9
**Total**		93	100

**Table 2:** Frequency and percentages of the effect size in the change in OHRQoL before and after GA.

Effect Size of OHRQoL	Frequency (*n*)	Percentage (%)
**Low effect size**	28	30.1
**Moderate effect size**	17	18.3
**Large effect size**	48	51.6
**Total**	93	100

**Table 3:** The association between different procedures under GA and the categories of the effect size of OHRQoL.

		Effect Size in the Change in OHRQoL			Total	*p*-Value *
		Low Effect Size	Moderate Effect Size	Large Effect Size		
**Extraction category**	extractions 3–4 teeth **	17	7	21	45	**0.32**
	extractions ≥ 5 teeth	11	10	27	48	
**Restoration category**	restorations < 3 teeth	15	11	27	53	**0.78**
	restorations ≥ 3 teeth	13	6	21	40	
**SSC category**	SSC < 4 teeth	11	6	21	38	**0.85**
	SSC ≥ 4 teeth	17	11	27	55	
**Pulp treatment category**	pulp < 2 teeth	15	11	21	47	**0.30**
	pulp ≥ 2 teeth	13	6	27	46	
**Total**		28	17	48	93	

* Using the Chi-square test, significant *p*-value < 0.05, ** Categorisation was performed based on mean score.

**Table 4:** Regression analysis between the effect of size on the change in OHRQoL, different socioeconomic factors, and different procedures.

Regression Analysis Using Effect Size of OHRQoL as Outcome		Exp (B)	*p*-Value
**Extraction category**	extractions 3-4 teeth **	0.98	**0.95**
	extractions ≥5 teeth	1	
**Restoration category**	restorations < 3 teeth	0.74	**0.21**
	restorations ≥ 3 teeth	1	
**Pulp treatment category**	pulp < 2 teeth	1.11	**0.62**
	pulp ≥ 2 teeth	1	
**Sex**		0.69	**0.12**
**Pain**		0.26	0.04 *
**Finance**		0.81	**0.17**

* Using linear regression model, significant *p*-value < 0.05. ** Categorisation was performed based on mean score.

## Data Availability

The original contributions presented in this study are included in the article. Further inquiries can be directed to the corresponding author.

## Abbreviations

The following abbreviations are used in this manuscript:

ECC	Early Childhood Caries
P-CPQ	Parental-Caregivers Perceptions Questionnaire
ECOHIS	Early Childhood Oral Health Impact Scale
KAUH	King Abdulaziz University Dental Hospital
ASA	American Society of Anaesthesiologists
OHBQ	Oral Health and Behaviour Questionnaire
ES	Effect size
SSCs	Stainless-steel crowns
AAPD	American Association of Pediatric Dentistry
CARIES	QC Child-Centered Caries-Specific Quality of Life
GA	General anaesthesia (GA)
OHRQoL	Oral health-related quality of life
A-ECOHIS	Arabic-Early Childhood Oral Health Impact Scale
FIS	Family Impact Scale

## References

[1] World Health Organization Oral Health. https://www.who.int/news-room/fact-sheets/detail/oral-health.

[2] Colak H., Dülgergil C.T., Dalli M., Hamidi M.M. (2013). Early childhood caries update: A review of causes, diagnoses, and treatments. J. Nat. Sci. Biol. Med..

[3] Ludovichetti F.S., Zuccon A., Cantatore D., Zambon G., Girotto L., Lucchi P., Stellini E., Mazzoleni S. (2022). Early childhood caries and oral health-related quality of life: Evaluation of the effectiveness of single-session therapy under general anesthesia. Eur. J. Dent..

[4] Mathew M.G., Jeevanandan G., Vishwanathaiah S., Hamzi K.A., Depsh M.A.N., Maganur P.C. (2023). Parental and child outlook on the impact of ECC on oral health-related quality of life: A prospective interventional study. J. Contemp. Dent. Pract..

[5] Orfali S.M., Alrumikhan A.S., Assal N.A., Alrusayes A.M., Natto Z.S. (2023). Prevalence and severity of dental caries in school children in Saudi Arabia: A nationwide cross-sectional study. Saudi Dent. J..

[6] Zafar S., Harnekar S.Y., Siddiqi A. (2009). Early childhood caries: Etiology, clinical considerations, consequences and management. Int. Dent. SA.

[7] Tinanoff N., Baez R.J., Diaz Guillory C., Donly K.J., Feldens C.A., McGrath C., Phantumvanit P., Pitts N.B., Seow W.K., Sharkov N. (2019). Early childhood caries epidemiology, aetiology, risk assessment, societal burden, management, education, and policy: Global perspective. Int. J. Paediatr. Dent..

[8] Farsi D.J., Farsi N.J., El-Housseiny A.A., Turkistani J.M., Farsi N.M. (2018). Impact of Dental Rehabilitation on Oral Health-related Quality-of-life in Healthy Children and Those with Special Health Care Needs. J. Contemp. Dent. Pract..

[9] El-Meligy O., Maashi M., Al-Mushayt A., Al-Nowaiser A., Al-Mubark S. (2016). The effect of full-mouth rehabilitation on oral health-related quality of life for children with special health care needs. J. Clin. Pediatr. Dent..

[10] American Academy of Pediatric Dentistry (2020). Behavior guidance for the pediatric dental patient. The Reference Manual of Pediatric Dentistry.

[11] Coté C.J., Wilson S., American Academy of Pediatrics, American Academy of Pediatric Dentistry (2019). Guidelines for monitoring and management of pediatric patients before, during, and after sedation for diagnostic and therapeutic procedures. Pediatrics.

[12] Sischo L., Broder H.L. (2011). Oral health-related quality of life: What, why, how, and future implications. J. Dent. Res..

[13] Pahel B.T., Rozier R.G., Slade G.D. (2007). Parental perceptions of children’s oral health: The Early Childhood Oral Health Impact Scale (ECOHIS). Health Qual. Life Outcomes.

[14] Jokovic A., Locker D., Stephens M., Kenny D., Tompson B., Guyatt G. (2002). Validity and reliability of a questionnaire for measuring child oral-health-related quality of life. J. Dent. Res..

[15] Bhoomika W., Munshi A. (2013). Relationship between severe early childhood caries and body mass index. J. Clin. Pediatr. Dent..

[16] Park J.S., Anthonappa R.P., Yawary R., King N.M., Martens L.C. (2018). Oral health-related quality of life changes in children following dental treatment under general anaesthesia: A meta-analysis. Clin. Oral Investig..

[17] Jankauskiene B., Virtanen J.I., Kubilius R., Narbutaite J. (2014). Oral health-related quality of life after dental general anaesthesia treatment among children: A follow-up study. BMC Oral Health.

[18] Chang J., Patton L.L., Kim H.-Y. (2014). Impact of dental treatment under general anesthesia on the oral health-related quality of life of adolescents and adults with special needs. Eur. J. Oral Sci..

[19] AlBader H. (2019). Changes in Oral Health Related Quality of Life in Children Following Dental Extractions Under a General Anaesthetic Using Two Child Self-Report Measures. Ph.D. Thesis.

[20] de Souza M.C., Harrison M., Marshman Z. (2017). Oral health-related quality of life following dental treatment under general anaesthesia for early childhood caries–a UK-based study. Int. J. Paediatr. Dent..

[21] Öztürk G., Gümüş H. (2024). Evaluation of oral health-related quality of life following dental rehabilitation under general anesthesia in Turkish children with early childhood caries. Int. J. Paediatr. Dent..

[22] Yawary R., Anthonappa R.P., Ekambaram M., McGrath C., King N.M. (2016). Changes in the oral health-related quality of life in children following comprehensive oral rehabilitation under general anaesthesia. Int. J. Paediatr. Dent..

[23] Alantali K., Al-Halabi M., Hussein I., El-Tatari A., Hassan A., Kowash M. (2020). Changes in preschool children’s oral health-related quality of life following restorative dental general anaesthesia. Br. Dent. J..

[24] Farsi N.J., El-Housseiny A.A., Farsi D.J., Farsi N.M. (2017). Validation of the Arabic Version of the Early Childhood Oral Health Impact Scale (ECOHIS). BMC Oral. Health.

[25] Abanto J., Tsakos G., Olegário I.C., Paiva S.M., Mendes F.M., Ardenghi T.M., Bönecker M. (2024). Impact of pulpectomy versus tooth extraction in children’s oral health-related quality of life: A randomized clinical trial. Community Dent. Oral Epidemiol..

[26] Pani S.C., Badea L., Mirza S., Elbaage N. (2012). Differences in perceptions of early childhood oral health-related quality of life between fathers and mothers in Saudi Arabia. Int. J. Paediatr. Dent..

[27] Farsi N., Ba’akdah R., Boker A., Almushayt A. (2009). Postoperative complications of pediatric dental general anesthesia procedure provided in Jeddah hospitals, Saudi Arabia. BMC Oral Health.

[28] Kirby J., Walshaw E.G., Yesudian G., Deery C. (2020). Repeat paediatric dental general anaesthesia at Sheffield Children’s NHS Foundation Trust: A service evaluation. Br. Dent. J..

